# Case of Diseased Brain

**Published:** 1805-09-01

**Authors:** Joshua E. White

**Affiliations:** Savannah, in the State of Georgia


### CASE OF DISEASED BRAIN; *by* DR. JOSHUA E. WHITE, *of Savannah, in the State of Georgia.*

[ Extracted from the New-York Medical Repository ]

ON the 21st of May last I was requested to see a mulatto boy, between seven and eight years old, who had been long sick, and of whose case I received the following history, as nearly as could be recollected by the mother.

In the summer of 1802 he had the measles in a favourable way, previous to which he had generally enjoyed good health. From this time he frequently complained of head-ach, would leave school in consequence of it, and had so constant a disposition to sleep, that it was with much difficulty he could be prevented from yielding to it, or be prevailed on to enter into the common amusements of children.

He was listless, inactive, and stupid. These symptoms continuing, in about two months he was attacked with convulsions at irregular periods. Almost every day he had one or more fits; and if sitting in a chair, would not fall, or, if near a wall, would support himself while it lasted, which was seldom more than two or three minutes. They generally terminated in sleep, for one, two, or three hours. The drowsiness continued to increase, and it was remarked to be always greater in the afternoon and evening. He would frequently cry without any apparent cause. His appetite, during this period, was irregular, sometimes voracious. About six months ago a swelling made its appearance about the junction of the right parietal with the occipital bone, which, gradually increasing, was, when I first saw him, about the size of two thirds of a billiard ball. It was soft, yielding, and elastic, and there was evidently a loss of substance in this part of the cranium. A smaller one was afterwards observed immediately behind the right ear, which had been noticed to increase slowly, while the other as gradually decreased, so as to induce a belief of a communication between the two. He was now observed to squint, the pupil of the right eye became dilated, and he gradually felt the motion of the right arm and leg to be diminished.

During the progress of these symptoms his head was noticed to grow much faster than any other part of his body; and a hat of the usual size for a boy of his age was much too small for him. It was, in fact, so remarkable, that among his play-mates he had the nick-name of big-head.

Upon my first visit, the most striking appearance which manifested itself was the unusual dimension of the head, particularly in a transverse direction. The pupil of the right eye constantly turned towards the nose, aud was less sensible than the other to the rays of light. His skin was generally hot, pulse quick and somewhat tense, thirst considerable, and bowels very costive; appetite impaired, and he had a constant propensity to sleep. The right side was paralytic; the arm and leg of that side were much less than of the other; and he had almost constant convulsive twitches in the right hand.

From the duration of his disease, the emaciated state of his system, and the symptoms denoting an organic affection of the brain, I had no hopes of a recovery. He was blistered; calomel was given in small doses, so as to affect the salivary glands; and it likewise acted briskly on the bowels. The salivation was continued for a fortnight, without effecting any change in the principal symptoms of his disease. There was now a discharge of puriform matter from the right ear, which, the mother says, possessed the singular quality of proving instantly fatal to flies which accidently lodged on it, insomuch that several have been found dead at one time within the concha and helix. He continued to have a return of convulsions almost every day, and in three weeks from the time of my first seeing him he expired.

Permission being obtained, an examination was made of the head on the morning after his decease, with the assistance of Dr. John Grimes.

From the upper part of one ear to the other, in a transverse direction, his head measured seventeen inches, and from the lower edge of the occipital to the same part of the frontal bone, sixteen inches.* After sawing through the cranium we found a firm adhesion of the dura mater to almost the whole inside of it, but particularly to the left parietal bone. The two anterior lobes of the brain were in a healthy state, but the left middle lobe, and the right posterior one, presented a remarkable appearance, totally different from the other contained parts. Perhaps no better idea can be formed of the change which had been produced by disease, than by comparing them to firm cream-coloured cheese. Between the cranium and the right posterior lobe was found some well-formed pus, and the skull was here thin and spongy. The ventricles contained about half a gill of dirty-coloured serum. In the other parts of the cerebrum, and in the cerebellum, no unusual appearances or marks of disease were discovered.

### REMARKS.

The diseased appearance of the two lobes which I have mentioned, evidently denoted them to be in a state of schirrosity; and this idea will gain additional weight by attending to the first symptoms and the progress of the disease. That the extraordinary induration was the effect of previous inflammation, I have not a doubt; and I am still more confirmed in the belief, from the very firm adhesion which was observed to have taken place between the cranium and dura mater, which we know can only arise from inflammation, and which we also know does not exist in health, except at the sutures and the basis of the scull.

It would seem, from the long continuance of the symptoms, that the inflammation partook of that kind which Dr. Cullen calls chronic, which by Dr. Brown has been called asthenic, and which has been named phrenicula by Dr. Rush.

---

* A more correct idea may be formed of the preternatural size of the head, by comparing the dimensions of his with those of a twin sister. From ear to ear her head measured twelve inches, and from the upper part of the nose to the inferior edge of the occipital bone, fourteen inches. Thus it will be observed that, in the first way, his head measured most, whereas in her's it measured the least.

Rush. The researches of the last-mentioned gentleman into the nature and causes of the internal dropsy of the brain, have been so far successful as to place the matter beyond a doubt, that this formidable disease is always the consequence of a primary inflammation in the brain, whatever may have been the direct or indirect causes of this inflammation.* The indirect cause of the congestion in the brain, in the above case, I believe to have been the measles.† As has been already noticed, he complained of his head from the period of his recovery from them; and perhaps the reason why water was not effused within the ventricles, and hydrocephalus formed, was owing to the less active nature of the inflammation than we know to be necessary for the effusion of water. A similar fact is often witnessed in other glandular parts of the body, particularly the liver and spleen; and is it not probable that the swelling of the joints in chronic rheumatism is the effect of a like action in the arteries of the part? It has also been remarked, that some pus was found between the cranium and the right posterior lobe; and for the formation of this, it is a fact well established, from the knowledge which we possess of the actions and powers of an animated body, that a certain degree of inflammation is indispensably requisite.

If the morbid appearances of the brain, on dissection, in the case just related, tend to elucidate the primary cause, and if the deductions I have drawn be admitted as correct, we shall have advanced a step further in our knowledge of that disease which has been generally known by the name of the internal dropsy of the brain, but which has been more properly called, by Dr. Rush, "chronic apoplexy." They will also aid in establishing the theory, as suggested by that gentleman and Dr. Quin; and, by attending to it, will consequently lead to a more successful practice than unfortunately has hitherto been exhibited in that formidable malady.

---

* Hence we see the impropriety of naming a disease hydrocephalus where no water is found on dissection.

† Several authors have mentioned instances of hydrocephalus consequent to eruptive diseases; and Dr. Rush says, he has seen a case in which it was evidently the consequence of the measles.

